# Whole brain 3D MR fingerprinting in multiple sclerosis: a pilot study

**DOI:** 10.1186/s12880-021-00620-5

**Published:** 2021-05-22

**Authors:** Thomaz R. Mostardeiro, Ananya Panda, Norbert G. Campeau, Robert J. Witte, Nicholas B. Larson, Yi Sui, Aiming Lu, Kiaran P. McGee

**Affiliations:** 1 Department of Radiology, Mayo Clinic, 200 1st St SW, Rochester, MN USA; 2 Department of Quantitative Health Sciences, Mayo Clinic, 200 1st St SW, Rochester, MN USA

**Keywords:** MR Fingerprinting, Multiple Sclerosis, Relaxometry, Normal appearing white matter, Splenium

## Abstract

**Background:**

MR fingerprinting (MRF) is a novel imaging method proposed for the diagnosis of Multiple Sclerosis (MS). This study aims to determine if MR Fingerprinting (MRF) relaxometry can differentiate frontal normal appearing white matter (F-NAWM) and splenium in patients diagnosed with MS as compared to controls and to characterize the relaxometry of demyelinating plaques relative to the time of diagnosis.

**Methods:**

Three-dimensional (3D) MRF data were acquired on a 3.0T MRI system resulting in isotropic voxels (1 × 1 × 1 mm^3^) and a total acquisition time of 4 min 38 s. Data were collected on 18 subjects paired with 18 controls. Regions of interest were drawn over MRF-derived T_1_ relaxometry maps encompassing selected MS lesions, F-NAWM and splenium. T_1_ and T_2_ relaxometry features from those segmented areas were used to classify MS lesions from F-NAWM and splenium with T-distributed stochastic neighbor embedding algorithms. Partial least squares discriminant analysis was performed to discriminate NAWM and Splenium in MS compared with controls.

**Results:**

Mean out-of-fold machine learning prediction accuracy for discriminant results between MS patients and controls for F-NAWM was 65 % (p = 0.21) and approached 90 % (p < 0.01) for the splenium. There was significant positive correlation between time since diagnosis and MS lesions mean T2 (p = 0.015), minimum T1 (p = 0.03) and negative correlation with splenium uniformity (p = 0.04). Perfect discrimination (AUC = 1) was achieved between selected features from MS lesions and F-NAWM.

**Conclusions:**

3D-MRF has the ability to differentiate between MS and controls based on relaxometry properties from the F-NAWM and splenium. Whole brain coverage allows the assessment of quantitative properties within lesions that provide chronological assessment of the time from MS diagnosis.

## Introduction

Multiple Sclerosis (MS) involves a wide spectrum of neurological symptoms resulting in challenging clinical management based on symptomatology alone [1]. Magnetic Resonance Imaging (MRI) has emerged as a powerful tool in the assessment of MS [2] with the requirement that imaging is performed using standardized imaging protocols [3].

With high diagnostic sensitivity, conventional MRI is able to describe disease dissemination in time and space [4], classify MS subtypes [2] and evaluate treatment response [2, 5]. However, conventional MRI may be limited when distinguishing ongoing inflammatory demyelinating pathology in normal-appearing white matter despite known disease processes [6] as well as functional disability [7].

It has previously been demonstrated that MS pathology can be described through quantitative spatial mapping of MRI-derived relaxometry parameters, such as longitudinal (T_1_) and transverse (T_2_) relaxation times or proton density (PD) [8]. Further, parametric mapping may overcome the aforementioned limitations associated with MS diagnosis and staging by improving diagnostic accuracy [6, 9, 10] and predicting patient functional impairment [11, 12].

MRF is a novel MRI technique that allows quantitative mapping of T_1_, T_2_ and PD using acquisition schemes followed by matching of the data to synthetically generated signals. The details of MRF have been described previously [13] and involve the repeated acquisition of image data over a time course in which acquisition parameters such as the flip angle, pulse repetition rate (TR) and echo time (TE) are intentionally modified [13]. Because the resultant time evolution of the signal in a given voxel is unique for a certain combination of tissue MR properties such as PD, T_1_ and T_2_, MRF derived estimates of these parameters are generated by comparing the signal evolution history of a given voxel to a dictionary of pre-simulated signal evolutions [14].

Acquiring brain relaxometry values in clinically feasible times in patients with MS have been proposed with the QRAP-MASTER pulse sequence [15]. This technique has yet to meet the requirement of being obtainable within a relatively short acquisition time and as a result has had limited application as part of a standard, time constrained clinical MR examination. MRF has the potential to address this time constrain and has been described as a promising classifier of MS subtypes [16] within imaging times of several minutes. However, in that work the acquisition involved only 2D data and did not provide full brain coverage, limiting the clinical use of such an approach.

The purpose of this study is to determine if MR relaxometry maps derived from a fast 3D-MRF executed as part of a standard clinical MR examination sequence can differentiate frontal lobe normal appearing white matter (F-NAWM) and splenium in patients with MS versus healthy volunteers based solely on MRF-based relaxometry differences. Further, we hypothesize that MRF can detect MS lesions and establish a temporal relationship between relaxometry values and the time since diagnosis.

## Materials and methods

### Image acquisition and reconstruction

All clinical data were acquired on two 3T MR scanners (Discovery MR750 and Discovery MR750W, GE Healthcare, Waukesha, WI) using an eight channel receive-only RF head coil. MRF data acquisition was performed using a 3D steady state free precession (SSFP) sequence with a multi-axis spiral trajectory [17]. Adiabatic inversion pulses were used before each acquisition. The flip angle ramped schedule ranged from 0.778° to 70°. Sequence details can be found in [17–19]. The acquisition FOV was 25.6 × 25.6 × 25.6 cm^3^ with 1mm isotropic voxel resolution. The total acquisition time for the whole brain volume was 4 min 38 s. The T_1_ range for the dictionary was from 10 to 3000 ms and T_2_ from 10 ms to 2000 ms. Fingerprint reconstruction and dictionary matching were performed offline using Matlab (Mathworks, Natick, Massachusetts) on a 64bit Linux workstation equipped with two 8‐core Intel Xeon Gold 6244 CPU @ 3.60 GHz, 376 GB system memory, and NVIDIA Tesla V100 GPU. The reconstruction pipeline has been described elsewhere [20].

### Patient population

An Institutional Review Board (IRB) approved protocol was used to obtain MRF data in patients scheduled for a clinical MR exam. Informed consent was obtained by all the participants. All methods were carried out in accordance with institutional guidelines and regulations. The MRF sequence was acquired during the clinical MRI prior to the administration of a gadolinium-based contrast agent. A total of 18 subjects with an established diagnosis of MS were included: 14 subjects had relapsing remitting MS, 3 had secondary progressive MS and 1 had primary progressive MS. Three subjects had active gadolinium enhancing MS lesions. In the control group, 18 subjects were selected and paired to age and gender for each individual with MS. Twelve of the 18 MS patients (mean age of 49 ± 13 years (mean ± SD)) were female. In the control group (n = 18; age mean ± SD age: 49 ± 14), 12 patients were female.

Time-since-diagnosis was defined as the time between the MRF exam and the earliest medical record clearly stating in the medical impression the patient had MS. To allow a better assessment of normal appearing white matter changes in MS, a wide range of distribution for time-since-diagnosis of MS was included. Out of the 18 subjects, 5 had been diagnosed with MS in less than 6 months. The time-since-diagnosis ranged from 1 to 270 months. The median time-since-diagnosis was 69 months (Percentile 25: 10 months; Percentile 75: 113 months). The mean ± SD time-since-diagnosis was 83 ± 79 months. The disease activity period (time since MS symptoms onset) largely corresponded to the time-since-diagnosis, except for 4 subjects who had MS disease activity months (range: 18–120 months) before the formal diagnosis.

### Regions of interest analysis

**Fig. 1:**
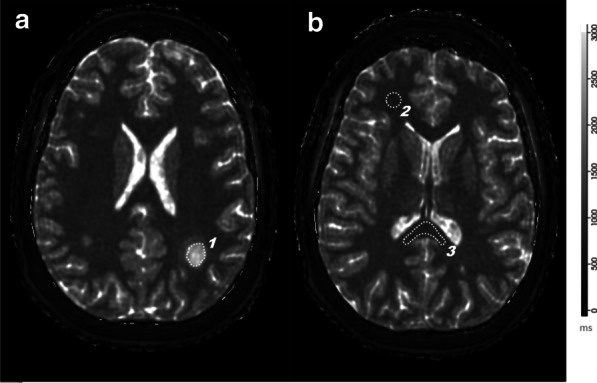
Region of Interest segmentations within MRF maps: MRF T_1_ relaxometry map demonstrating an active lesion in a patient with multiple sclerosis. **a** Depicts the lesion [1] in the parietal white matter and the corresponding ROI. The T_1_ and T_2_ values and the first order statistics were simultaneously obtained from this ROI. **b** shows the ROI over the frontal normal appearing white matter [2] and splenium [3]

Segmentations were performed manually using 3D-Slicer software [20] as described in Fig. 1. Four to ten lesions were selected for each patient with MS, with a total of 105 lesions across 18 patients, 10 of which were active lesions. Perilesional edema was not included in the segmentation of active lesions. Additionally, for each patient, one ROI each in F-NAWM and splenium of the corpus callosum were drawn. F-NAWM was defined as areas without signal changes on the standard T2 weighted images in the clinical exam. In the control group, corresponding ROIs were drawn in the F-NAWM and splenium. First order statistics (interquartile range, skewness, uniformity, median, energy, robust mean absolute deviation, mean absolute deviation, total energy, maximum, root mean squared, 90 percentile, minimum, entropy, range, variance, 10 percentile, kurtosis, mean) obtained from each ROI were analyzed. All segmentations were reviewed by a Board certified neuroradiologist.

#### Statistical analysis

Distributional characteristics of categorical variables were summarized as counts and percentages, while quantitative values were summarized by means and standard deviations (SD) or medians and quartiles where indicated. Given the paired nature of the study design, univariate statistical comparisons between cases and controls were made for all individual relaxometry features (interquartile range, skewness, uniformity, median, energy, robust mean absolute deviation, mean absolute deviation, total energy, maximum, root mean squared, 90 percentile, minimum, entropy, range, variance, 10 percentile, kurtosis, mean) using two-sided non-parametric Wilcoxon signed-rank tests. Visualization of multivariate relaxometry data was performed using unsupervised t-distributed stochastic neighbor embedding (t-SNE) dimensionality reduction based on two components under default settings. These visualizations were performed separately by T_1_ and T_2_ feature set as well as combined. Multivariate discrimination analysis between cases and controls for F-NAWM and splenium ROIs was performed using a two-component multi-level sparse partial least squares discriminant analysis (sPLS-DA) as implemented in the mixOmics R package [21]. These supervised machine learning analyses used a combined feature set from T_1_ and T_2_ relaxometry. Discrimination performance was characterized using area under the receiver operating characteristic curve (AUC) based on leave-one-out cross-validation along with corresponding p-values based on Wilcoxon testing. Univariate discrimination of MS lesions from F-NAWM and splenium using first order statistics features of centrality (mean and median) was evaluated using clustered ROC analyses to account for intra-patient correlation. Patient-level correlation testing between T_1_ and T_2_ relaxometry values and time since diagnosis for MS cases was performed using the nonparametric Kendall’s tau rank correlation test, using the mean value across lesions for a given patient. Primary analyses focused on T1 and T2 lesion means, with exploratory analyses expanded out to all regions and feature types. All analyses were performed using the statistical software R v3.6.2, and all reported p-values are unadjusted for multiple testing.

## Results

**Fig. 2:**
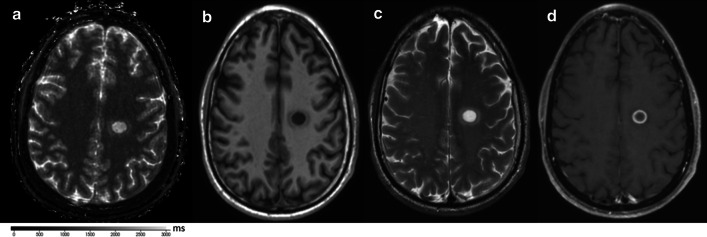
Active lesion with surrounding edema in a patient confirmed with multiple sclerosis: **a** depicts the lesion in the parietal white matter on MRF-based T_1_ map; **b** shows the same lesion in the same clinical exam in a T_1_ weighted sequence, **c** illustrates the typical high intensity on a T_2_ weighted spin echo sequence, and **d** confirms peripheral enhancement after gadolinium injection in a T_1_ weighted sequence.

A representative MRF-based T_1_ map paired with conventional weighted imaging is shown in Fig. 2.

**Fig. 3:**
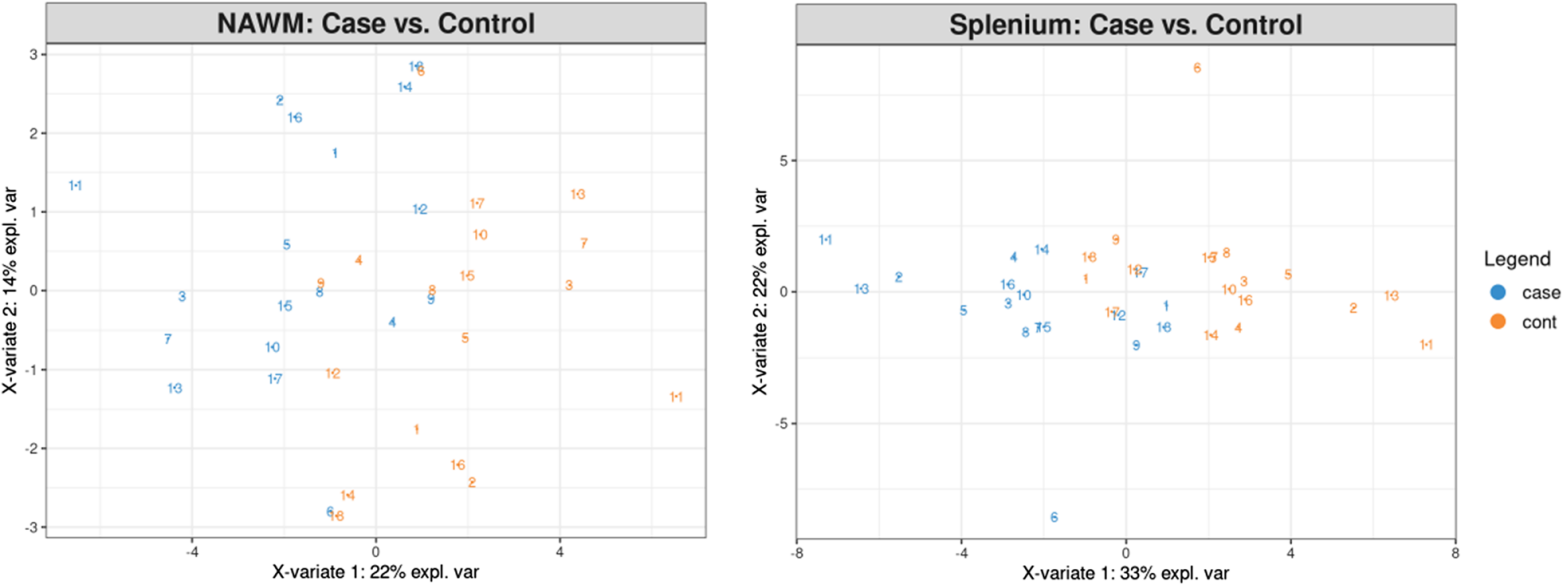
Frontal NAWM and Splenium classification for MS compared to control: Partial least squares discriminant analysis between patients (cases) and controls for splenium and frontal NAWM (normal appearing white matter) within all 18 MS patients and 18 controls

Partial least squares discriminant analysis (PLS-DA) for F-NAWM and splenium is displayed in Fig. 3. Repeated cross validation (n = 5) showed mean out-of-fold accuracy = 65 % (AUC = 0.625 (p = 0.21)) for discriminant results between patients and controls for F-NAWM, but mean out-of-fold accuracy approaching 90 % (AUC = 0.880, p < 0.0001) for splenium, primarily via component 1. Examination of the component 1 feature loadings indicated maximum T1 value (-0.30) and T1 robust mean absolute deviation (-0.28). This was commensurate with univariate findings, where the top associations (Table 1) corresponded to T1 distributional measures of extrema (e.g., T1 90 % percentile: Wilcoxon p = 0.002) and variability (e.g., T1 Root Mean Squared: Wilcoxon p = 0.003).

**Table 1:** Most significant individual relaxometry features allowing differentiation between splenium and frontal normal appearing white matter in multiple sclerosis

Relaxometry	Region	Feature	p value
T1	Splenium	90 percentile	0.002*
T1	Splenium	Root mean squared	0.003*
T1	Splenium	Mean	0.003*
T1	Splenium	Robust mean absolute deviation	0.005*
T1	Splenium	Median	0.005*
T1	Splenium	Interquartile range	0.006*
T1	Splenium	Mean absolute deviation	0.007*
T1	Splenium	Entropy	0.010*
T1	Splenium	Uniformity	0.012*
T2	Splenium	90 percentile	0.022*

*p < 0.05 connotates statistical significant. Two-sided non-parametric Wilcoxon signed-rank tests

**Fig. 4:**
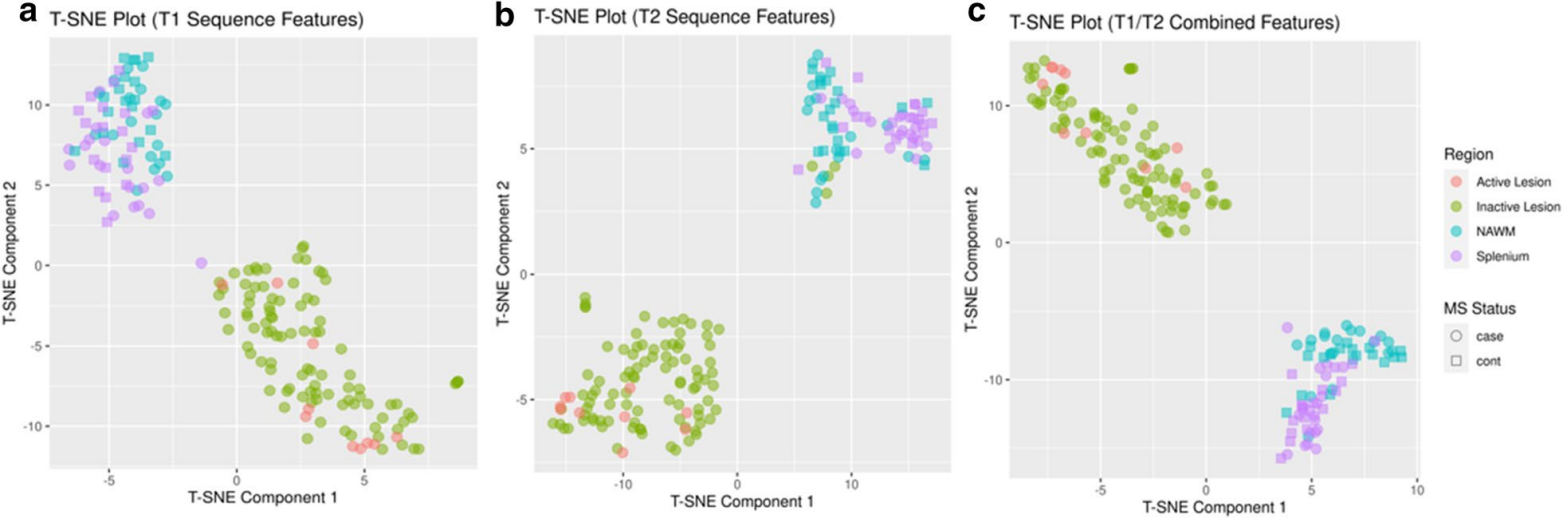
T-distributed stochastic neighbor embedding clustering for all segmentations: these figures demonstrate strong clustering of the data under a T-SNE algorithm, and this allows apparently perfectly discrimination between lesions and non-lesions with T_1_ (**a**) T_2_ (**b**) and combining the two properties (**c**) features. NAWM: frontal normal appearing white matter; MS: Multiple Sclerosis. The data depicted in circles refers to patients with MS and the data on square to controls

**Fig. 5:**
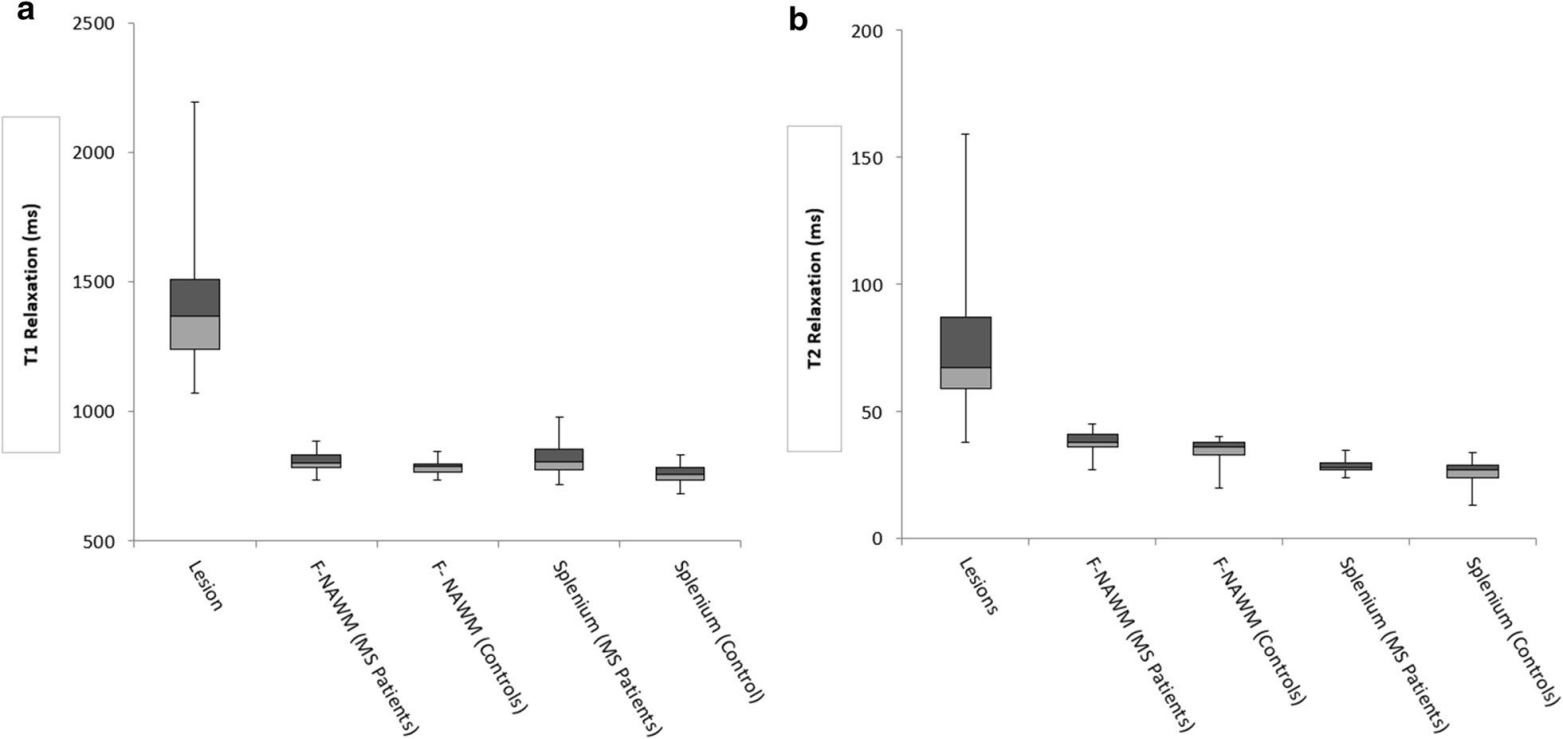
Box and whisker plots for differences in T1 (**a**) and T2 (**b**) mean relaxation times between the structures analyzed. The vertical lines depict the ranges, the light boxes the second quartile, the dark gray boxes the third quartile and the solid vertical line the median. For the lesions, the 25th percentile (T1; T2: 1240 ms; 59 ms), median (T1;T2: 1368 ms; 67 ms), 75 percentile (T1;T2: 1509 ms; 87 ms) were higher compared to all the anatomical structures. The T1 and T2 scales are on milliseconds (ms). F-NAWM: frontal appearing normal white matter

The T-SNE Plot for classification of MS lesions is displayed on Fig. 4. AUC analysis for selected features demonstrated that median and mean T_1_ and T_2_ allowed perfect discrimination (AUC = 1) between splenium and lesions for both T_1_ and T_2_. Also, discrimination from F-NAWM was excellent (AUC = 1) and (AUC = 0.98) using median and mean for T_1_ and T_2_, respectively. Figure 5 depicts the distribution range of mean T_1_ and T_2_ relaxometry ranges for all structures analyzed.

**Fig. 6:**
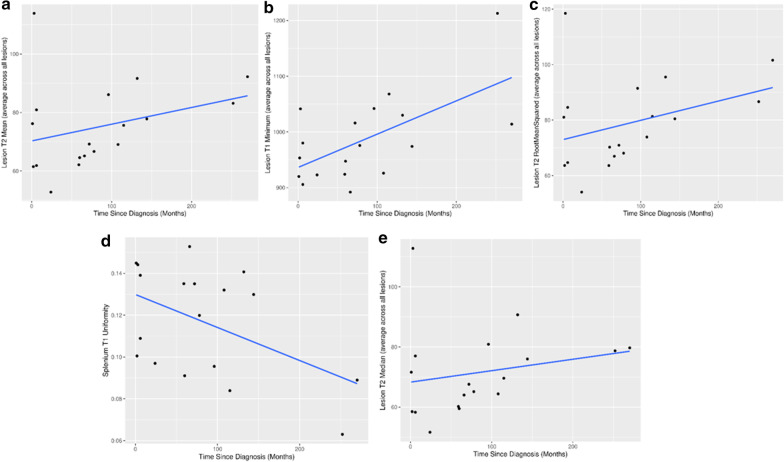
Visualization of the top 5 features correlated with time-since-diagnosis. The correlation was the strongest for **a** T2 lesion mean (rho = 0.419, p = 0.015), **b** T1 lesion minimum (rho = 0.367, p = 0.033), **c** T2 lesion root mean squared (rho = 0.367, p = 0.033), **d** T1 splenium uniformity (rho = −0.354, p = 0.04) and **e** T2 lesion median (rho = 0.354, p = 0.040). Multiple lesions are collapsed to a singular value via arithmetic mean

Correlation analyses among lesion means and time-since-diagnosis for MS cases was yielded higher rank correlation estimates for T2 (rho = 0.419, p = 0.015) than T1 (rho = 0.257, p = 0.11). The top five rank correlations among all relaxometry features and time since diagnosis are presented in Fig. 6.

## Discussion

This work describes the use of a novel whole brain 3D MRF sequence [17, 18] in differentiating F-NAWM and splenium in patients with MS based on relaxometry estimates. Given the highly reproducible and accurate information provided by MR relaxometry [22, 23] the results of this study, and the isotropic whole brain coverage afforded by this technique, MRF has the potential for use in the diagnosis of patients with MS. The previously described application of MRF in the normal brain [22, 24], brain tumors [25, 26], epilepsy [27] and Parkinson disease [28], suggests that MRF has the potential for even broader application beyond MS.

MRI currently is a fundamental clinical tool when guiding therapy for patients with MS [29]. Given the complexity of the condition, several studies have been conducted with more advanced MRI techniques (such as myelin water fraction or functional MRI) to predict whether MS could be diagnosed by machine learning techniques [30–33]. Although the mentioned investigations have been successful, those techniques differ from MRF in that they do not provide a multi parametric approach from a single acquisition leading to lengthier exam acquisitions. Furthermore, the reproducibility of said techniques is not as well established as MRF-based relaxation estimates for both in vivo and phantom experiments [23, 34].

F-NAWM demonstrated longer relaxation in patients with MS in our study. This has been described by other quantitative imaging investigations [35]. Those changes are thought to be related with myelin histological changes in the white matter poorly defined by imaging [36] and importantly could predict clinical disability [12]. In this study, F-NAWM differentiation using MRF relaxation properties between cases and controls was fairly weak (mean out of fold accuracy = 65 %). Given the moderate sample size, it is possible larger samples could describe more robust differentiation. Also, it is important to note prior studies [35, 37, 38] have provided estimates of the entire NAWM through the brain, potentially including areas adjacent to MS plaques that can have subtle signal changes. In order to avoid this pitfall, values stated in this work were from segmented areas that only included white matter with no changes in the conventional T_2_ weighted imaging and double inversion recovery.

Splenium is partially responsible for interhemispheric connections within the brain [39]. As such, studies describing splenium changes in patients with MS [40] have focused on diffusion tensor imaging. However, histological changes in MS may also be responsible for relaxation lengthening in the splenium [35]. This could explain why ROI features related with a longer relaxation such as the percentile, mean and median were the most important in the differentiation of MS from controls in our study. The accuracy described in this study for classifying disease and control at this anatomical site based solely on MRF-based relaxometry changes was fairly strong (= 90 %), identifying a major advantage of MRF. Given its potential to depict changes that are currently not seen or described in clinical practice, MRF may be useful, especially in those cases where the diagnosis of MS is not clearly established by more conventional well-established imaging protocols.

It is known that time since diagnosis in MS can influence normal tissue relaxation [7, 11, 38], and that those changes could predict clinical disability [41, 42]. Papadoulos et at [7] described NAWM relaxation changes in a longitudinal study covering 5 years. However, Davies et al. [11] found no significant differences in a three year longitudinal study after accessing T_1_ quantitative changes through NAWM and GM. In this study, T_2_ lengthening was observed in MS plaques on those patients with the longest time from diagnosis of MS to imaging. These findings could be related to a higher degree of Wallerian degeneration [43] although this finding has questionable clinical significance. Also, given this study was cross sectional, it would be valuable to investigate MRF through the same protocol in a longitudinal basis, so F-NAWM and splenium changes may be described and the faster acquisition as compared with the protocols mentioned [7, 41, 42] could be a valuable tool for clinical application. Importantly, this investigation described the diagnosis of MS as the surrogate for the disease duration. As such, given the onset of MS symptoms was before the time of the MS diagnosis for 4 volunteers (in a selected case by several years), the effects of disease activity before the diagnosis were not well described by our investigation.

This study has several limitations. The relatively small sample size may not be sufficient to effectively establish F-NAWM and splenium changes in MS as compared to controls. Also, F-NAWM segmentations represented a minimal fraction of the overall WM in all the patients included. Both active and non-active lesions were included, as defined by gadolinium enhancement in conventional T1 weighted imaging, but given only 10 lesions were active, this study was not powered to detect changes within relaxometry for classifying lesion activity. Future studies with larger sample sizes and volumetric segmentation through normal appearing white matter may be considered.

## Conclusions

3D-MRF relaxation changes in the splenium and to a lesser degree in the F-NAWM were able to discriminate the presence of MS disease as compared to controls. Those findings corroborate the potential clinical role of MRF relaxometry where suspicious white matter changes are present, as MRF could either support or counter the presumptive diagnosis. Furthermore, quantitative evaluation of MRF derived relaxometry was helpful in characterizing the chronicity of the demyelinating lesions.

## Data Availability

The datasets used during the current study are available from the corresponding author on reasonable request.
