# Correction to: Whole brain 3D MR fingerprinting in multiple sclerosis: a pilot study

**DOI:** 10.1186/s12880-021-00673-6

**Published:** 2021-09-27

**Authors:** Thomaz R. Mostardeiro, Ananya Panda, Norbert G. Campeau, Robert J. Witte, Nicholas B. Larson, Yi Sui, Aiming Lu, Kiaran P. McGee

**Affiliations:** 1 Department of Radiology, Mayo Clinic, 200 1st St SW, Rochester, MN USA; 2 Department of Quantitative Health Sciences, Mayo Clinic, 200 1st St SW, Rochester, MN USA

## Correction to: BMC Med Imaging (2021) 21:88 10.1186/s12880-021-00620-5

Following the publication of the original article [1], the authors requested to include the following “Acknowledgements” section that had initially been omitted:

“The authors would like to acknowledge the support of GE Healthcare and the University of Pisa for proving the MR fingerprinting sequence and analysis software”.

The original article has already been corrected as above.
